# Real‐Time Imaging of the Mechanobactericidal Action of Colloidal Nanomaterials and Nanostructured Topographies

**DOI:** 10.1002/smsc.202300002

**Published:** 2023-04-05

**Authors:** Felipe Viela, Ingrid V. Ortega, Jaime J. Hernández, Isabel Rodríguez, Sara Moreno-Da Silva, Alejandro López-Moreno, Emilio M. Pérez, Cristina Flors

**Affiliations:** ^1^ Madrid Institute for Advanced Studies in Nanoscience (IMDEA Nanociencia) C/ Faraday 9 28049 Madrid Spain; ^2^ Nanobiotechnology Associated Unit CNB-CSIC-IMDEA C/ Faraday 9 28049 Madrid Spain

**Keywords:** bacteria/material interfaces, carbon nanotubes, fluorescence microscopy, mechanobactericidal strategies, Min oscillations, nanofabricated topographies, real-time imaging

## Abstract

Mechanobactericidal nanomaterials, such as low‐dimensional nanoparticles in suspension or high‐aspect‐ratio nanofabricated topographies, rely on their mechanical or physical interactions with bacteria and are promising antimicrobial strategies that overcome bacterial resistance to classical antibiotics. However, the underlying killing mechanisms are poorly understood, given the challenges associated with the real‐time characterization of the mechanical interaction in a biologically relevant environment. Indeed, different death mechanisms have been proposed depending on the magnitude of the interaction forces. Herein, the real‐time and single‐cell response of bacteria to weak mechanical interactions with nanostructured topographies and “nanodarts,” exemplified by flowing single‐walled carbon nanotubes, is investigated. To that end, an advanced reporting strategy is used to follow sublethal physiological effects on bacteria upon contact with these materials. With this method, it is estimated that the contact time at which the initial stages of bacterial death occur is in the order of a few tens of minutes. This information contributes to a full understanding of the complex mechanisms of mechanically induced bacterial death.

## Graphical abstract

The real‐time response of bacterial cells to mechanical interactions with mechanobactericidal materials is investigated. To that end, an advanced reporting strategy is used to follow sublethal physiological effects on bacteria upon contact with mechanobactericidal materials. This method is able to estimate the contact time at which an initial detrimental physiological response occurs.

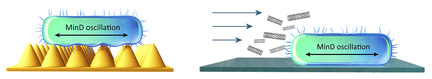

## 1 Introduction

The past decade has seen the emergence of novel antibacterial strategies that rely on mechanical or physical interaction between nanomaterials and bacteria.^[^ ^1^ ^]^ These “mechanobactericidal” nanostructures can be low‐dimensional nanoparticles in suspension or high‐aspect‐ratio topographies that are nanofabricated on surfaces. A clear advantage of mechanobactericidal strategies compared to traditional antibiotics is that bacteria are not able to evolve resistance to the former. However, there is a debate about the processes that lead to the mechanobactericidal action of these nanostructures.^[^ ^2^ ^]^ Indeed, fundamental aspects of the physical interaction between bacteria and nanostructures, and the underlying killing mechanisms, are poorly understood. This is in part due to the challenges associated with the real‐time characterization of this interaction in a biologically relevant environment.

Computational and experimental methods have contributed so far to understand some aspects of the mechanobactericidal activity of nanomaterials. Modeling studies have proposed that the topographical nanofeatures induce the critical deformation of the bacterial membrane as a consequence of excessive local stretching.^[^ ^3^ ^]^ There is still debate in the literature about the nature and the magnitude of the underlying forces that may produce this effect, for example, spontaneous adhesion of the cell walls to the nanostructures,[^3^, ^4^] shear and capillary forces,^[^ ^5^ ^]^ or storage and release of mechanical energy.^[^ ^6^ ^]^ Moreover, recent studies suggest that other death mechanisms are possible without membrane penetration of the nanostructures, for example, by stress responses that lead to high levels of reactive oxygen species (ROS),^[^ ^7^ ^]^ impaired metabolic activity,^[^ ^8^ ^]^ or altered genomic and proteomic profile.^[^ ^9^ ^]^ A similar interplay between direct membrane piercing and mechanical fatigue has also been proposed for low‐dimensional nanomaterials in suspension.^[1a]^ It is very likely that the precise mechanism will depend on the geometry or mechanical properties of the nanomaterial.[^1^, ^6^] Understanding the death mechanisms and quantifying the forces involved can be extremely valuable to design more efficient mechanobactericidal materials.

Our laboratory has recently combined fluorescence and atomic force microscopy (AFM) to quantify the forces necessary to inflict mechanical damage on a single bacterial cell.^[^ ^10^ ^]^ In this study, an AFM tip was used as a simplified model of a high‐aspect‐ratio nanostructure, and complementary fluorescence labeling strategies were used to probe the bacterial response to external mechanical force in real‐time. The required force for the mechanical rupture of the bacterial cell wall in *Escherichia coli* was estimated as 20 nN, which separates two regimes of interaction: one at higher force that leads to instant bacterial death and another one that characterizes low force collisions between bacteria and nanomaterials. The study found that the latter may also lead to bacterial death by fatigue effects. The value of 20 nN for cell wall penetration and instant death was higher than expected, supporting the notion that bacterial death mechanisms that do not involve cell wall rupture are relevant to fully understand the action of mechanobactericidal materials.[^2^, ^8^] Herein, we explore in more detail the effect of weak mechanical interactions on bacterial response that eventually lead to death by mechanical fatigue. To understand more globally the effect of mechanobactericidal nanomaterials, we include nanostructured topographies and “nanodarts,” exemplified by flowing single‐walled carbon nanotubes (SWCNTs).^[1a]^ While the bactericidal properties of both types of nanomaterials are well established, we aim at providing a clearer picture of the influence of mechanical effects in their killing mechanism, especially the timescale at which weak mechanical interactions produce a significant response on bacteria. To that end, we investigate the effect of these nanomaterials in real‐time and at the single‐bacterial level using suitable fluorescence microscopy strategies. As in our previous work combining fluorescence microscopy and AFM nanoindentation,^[^ ^10^ ^]^ fatigue effects in bacteria are assessed by following the oscillations of the Min protein system as a reporter. The Min system is a cell division regulator in *E. coli*. Under normal growth conditions, MinD is associated with the membrane and undergoes pole‐to‐pole oscillations.^[^ ^11^ ^]^ The period of these oscillations has been previously proposed as a reporter for bacterial physiological state at the single‐cell level, and it is likely related to membrane potential dynamics.^[^ ^12^ ^]^ The changes in the Min oscillation period have been used to monitor the response to sublethal challenges from antibiotics, temperature, mechanical fatigue, or photodynamic treatment on *E. coli*.^[^ ^10^, ^13^ ^]^ Our real‐time microscopy results are complementary to other theoretical and experimental approaches, since they are focused on the physiological response of live bacteria to the interaction with nanomaterials.

## 2 Results and Discussion

### 2.1 Real‐Time Imaging of Bacterial Response to Nanostructured Topographies

**Figure 1:**
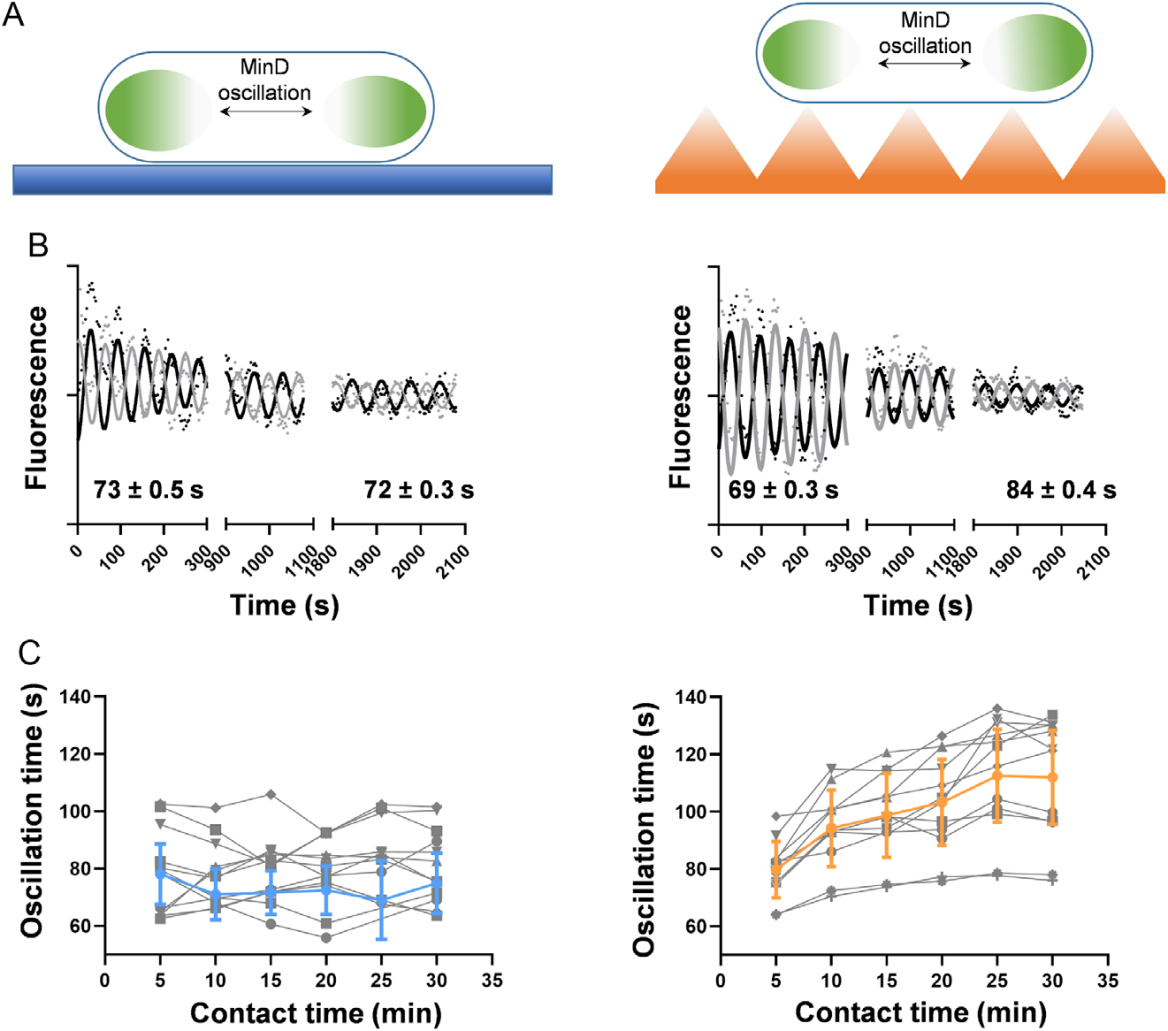
Real‐time monitoring of mechanically induced variations of MinD oscillation period at single‐cell level on OrmoComp surfaces. A) Representation of the experimental approach to study the variations of GFP‐MinD oscillations on different surfaces. B) Pole‐to‐pole oscillation of GFP‐MinD of *E. coli* attached to flat (left) and nanostructured (right) surfaces. Black and grey dots represent the fluorescent intensity on each pole over time. Black and gray lines correspond to the damped sinusoidal fitting; C) Representation of the variation of MinD oscillation period versus the contact time with flat (left) and nanotopographies (right) for different cells. The blue and orange lines represent the mean values; the whiskers correspond to the standard deviation. The gray lines represent the oscillation times for individual cells.

The optically transparent UV‐curable resin OrmoComp was used to fabricate the nanostructured topographies to allow for good optical transparency during single‐cell fluorescence microscopy. The fabricated nanotopographies, which are bioinspired from the moth's eye,^[^ ^14^ ^]^ were characterized by scanning electron microscopy (see Figure S1, Supporting Information). A well‐defined array of nanocones with a mean height of 350 nm, width of 80 nm at the tip, and an aspect ratio of 4.3, arranged on a dense hexagonal lattice of 250 nm pitch, was observed. It was previously shown that these same nanostructures fabricated on poly (methyl methacrylate) are able to kill up to 40% of Gram‐negative bacteria after they were in contact for 1–7 h.[^14^, ^15^] We aimed at monitoring the real‐time response of *E. coli* in contact with the nanostructured topographies at shorter times by using Min oscillations as a reporter, and compare it with the response to contact with a flat OrmoComp surface (**Figure** **1**A). Thus, bacteria were allowed to interact during 5 min with both surfaces, which were treated with a low concentration of poly‐L‐lysine (PLL) to promote bacterial attachment (see Experimental Section), and fluorescence movies were collected at 5 min intervals during a total contact time of 30 min (Movie S1, Supporting Information). At the single‐bacterial level, the oscillation of GFP‐MinD between both poles produces fluorescence traces that can be fitted with a damped sinusoidal function, to account for fluorescence photobleaching (see Figure 1B and Experimental Section). Each curve is characterized by an oscillation period, which is expected to increase (i.e., the oscillation becomes slower) when bacterial physiology is compromised.^[13b,13d]^ For the bacterium on the flat surface, the oscillation period after 30 min of contact time is essentially the same as at time zero, suggesting no effect on bacterial physiology. In contrast, contact with the nanostructured topography slows down the oscillation period from 69 to 84 s, confirming that the bacterium is affected already after 30 min of contact time. A more comprehensive analysis representing the dependency of the oscillation period versus the contact time for several individual bacteria (Figure 1C) shows a progressive slowdown of MinD oscillation for bacteria adhered to the nanotopographies, while it stays constant on bacteria attached to flat surfaces (Figure 1C).

**Figure 2:**
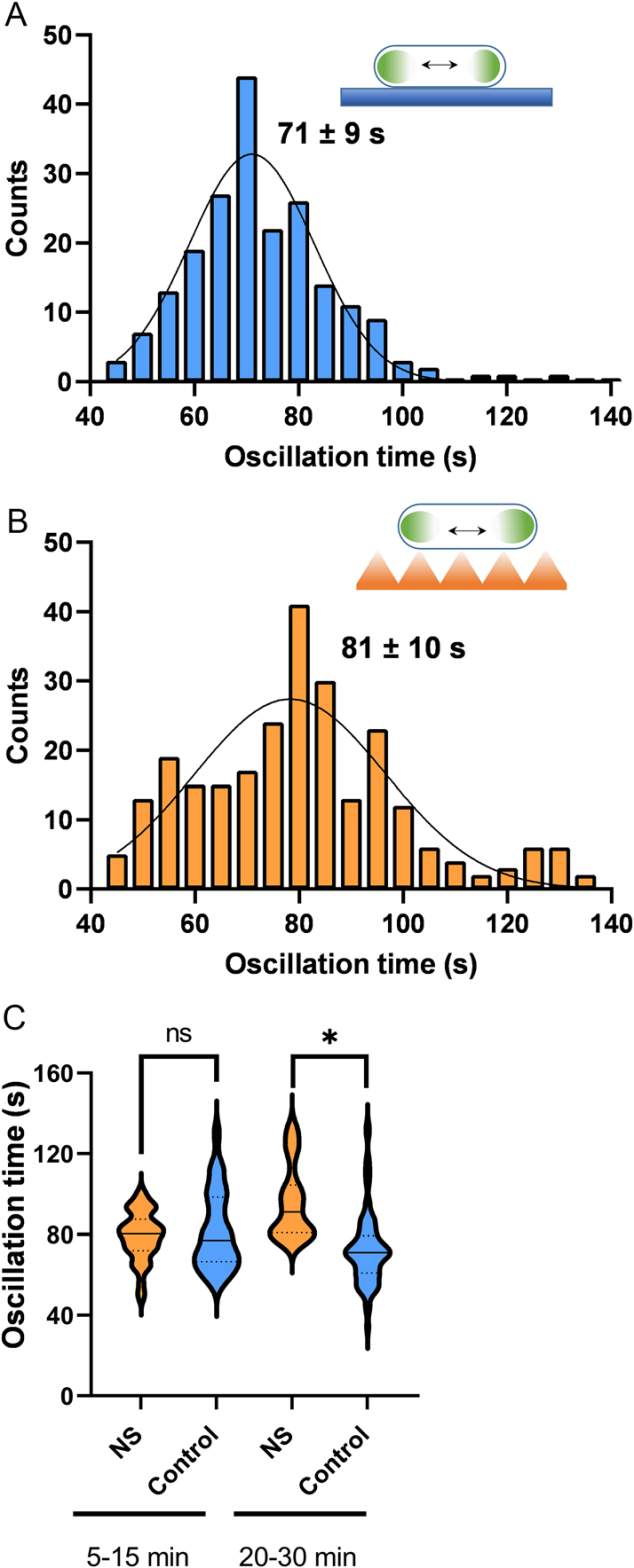
Impact of polymer nanostructured topographies (NS) on bacterial physiology. A) Distribution of oscillation periods of bacteria immobilized on flat topographies, *n* = 165. B) Distribution of oscillation periods of bacteria immobilized on NS, *n* = 175. C) Distribution of MinD oscillation data at two different time ranges. The solid line represents the mean value, dashed lines represent the first and third quartiles (*p < 0.05).

**Figure** **2** shows a histogram collecting the single‐cell behavior over time of tens of bacteria with up to 30 min of contact time with the flat surface, with an average oscillation period of 71 ± 9 s (mean ± SD). In comparison, bacteria that were in contact with a nanostructured surface for the same time showed a longer average oscillation period of 81 ± 10 s.

To study in more detail the timescale of the observed physiological response, data points in the histograms in Figure 2A,B were divided into two groups, the first one defined as an initial contact from 5 to 15 min and the second one from 20 to 30 min that corresponds to a more established interaction. Violin plots in Figure 2C show data segregated by contact time and suggest that a significant increase in the Min oscillation period occurs after about 20 min on nanotopographies in comparison with the flat surface control.

It is worth noting that after 140 min of contact time, only about 10% of bacteria were stained by the viability indicator propidium iodide (PI), which informs about membrane integrity (Figure S2, Supporting Information). This is consistent with a previous study in which 7 h of incubation were necessary to reach about 40% killing,^[14a]^ and it highlights that using Min oscillations as a reporter is useful for imaging the initial stages of bacterial death on nanofabricated topographies. As previously discussed,^[^ ^10^, ^13^ ^]^ Min oscillations are able to report subtle physiological effects that are overlooked by viability indicators such as PI, emphasizing the complementarity of both methods.

### 2.2 Real‐Time Imaging of High‐Speed Collisions of “Nanodarts” on Bacteria

**Figure 3:**
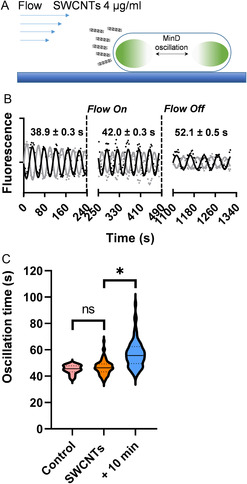
Impact of SWCNTs on bacterial physiology under mechanical interaction promoted by flow. A) Experimental approach to evaluate the impact of SWCNTs on bacterial physiology under flow. B) Real‐time tracking of the variation of MinD oscillation period during and after flow of SWCNTs for a single bacterium. C) Comparison of the oscillation period of MinD, before, during, and after SWCNTs flow for *n* = 78 (*p < 0.05).

Dispersed SWCNTs can be visualized as moving “nanodarts” in solution, producing a detrimental effect on bacterial physiology and integrity. Previous work has shown that the major cause for bacterial death is physical interaction or puncture, as suggested by a higher bactericidal efficacy of individually dispersed SWCNTs compared to aggregates, or of higher shaking speeds during incubation.^[^ ^16^ ^]^ Moreover, it has been suggested that this bactericidal activity is the result of the accumulation effect of a large number of collisions.^[^ ^17^ ^]^ Here, we follow this process in real‐time and at a controlled flow rate of dispersed SWCNTs. To that end, a microfluidic system connected to a syringe pump was used to produce high‐speed collisions between immobilized bacteria and flowing SWCNTs (**Figure** **3**A). First, *E. coli* immobilization conditions were optimized to find a balance between strong adhesion (to withstand flow) and impact of immobilization on their physiological properties (assessed by following changes in the oscillation period of GFP‐MinD). We found that mild immobilization by incubating bacteria for 20 min on surfaces coated with 0.01% PLL is a good compromise between these parameters. Live bacteria could sustain a flow of PBS and 0.3% Tween 20 (a biocompatible surfactant that will be used later to suspend SWCNTs) at a rate of 3.7 mL min^−1^, corresponding to a shear stress of about 12 dyn cm^−2^ (see Experimental section and Figure S3, Supporting Information). We note that for these experiments, *E. coli* DH10β was used, since this strain produced higher GFP fluorescence intensity, necessary for these more challenging experiments under flow. It has been previously reported that Min oscillation periods are dependent on the *E. coli* strain,^[^ ^18^ ^]^ and we found that DH10β shows a shorter average Min oscillation period compared to the BL21 strain used above. Once these conditions were established, SWCNTs (6,5) chirality, 0.78 nm average diameter at a concentration of 4 μg mL^−1^, were dispersed in PBS with Tween 20 at 0.3% by sonication and centrifugation (see Experimental Section and Figure S4, Supporting Information). Before flowing the SWCNT suspension into the microfluidic chamber, immobilized bacteria were first imaged for 4 min to record the oscillation period of GFP‐MinD as a control (Figure 3B). Flow was then initiated at 12 dyn cm^−2^ during 4 min, and afterward the flow was stopped and the effect on Min oscillation was observed after 10 more min (Movie S2A and S2B, Supporting Information). For the bacterium imaged in Figure 3B, a clear lengthening of the oscillation period is found at the end of the experiment. Violin plots collecting the individual behavior of tens of bacteria show that, while no significant change in the Min oscillation period was observed during flow, the period of Min oscillations increased significantly to 55.1 ± 9.1 s after the 10 min incubation (Figure 3C). To verify that the observed effect can be attributed mainly to the enhanced mechanical interaction promoted by the flow of SWCNTs, we performed the same experiment under static conditions (Figure S5, Supporting Information). Indeed, purely static incubation with SWCNTs did not produce an observable change in the oscillation and in turn, bacterial physiology. These results showcase again the usefulness of Min oscillations to follow sublethal mechanical interactions between nanostructures and bacteria.

## 3 Conclusion

We have explored in real‐time and at the single‐cell level the effect of weak mechanical interactions between bacterial cells and mechanobactericidal nanomaterials. These nanomaterials are exemplified by surface nanotopographies, which are fabricated using a polymer with good optical properties compatible with single‐cell imaging, as well as flowing SWCNTs in solution. We show that Min oscillations are a useful tool to study this range of sublethal mechanical interactions, since they report early physiological changes in *E. coli* that are overlooked by viability dyes such as PI.^[^ ^10^, ^13^ ^]^ For both mechanobactericidal materials studied here, the evolution of Min oscillation behavior informs about the timescale at which bacterial physiology starts to be compromised by mechanical interaction, which is in the order of a few tens of minutes. This time range is relevant for the activation of stress responses such as ROS formation or metabolic changes that may eventually lead to bacterial death.^[^ ^19^ ^]^ Overall, this information contributes to a full understanding of bacteria–nanomaterial interactions and the complex mechanisms of mechanically induced bacterial death, and may provide design guidelines for the next generation of mechanobacteridical nanomaterials.

## 4 Experimental Section

4.1

4.1.1

##### Bacterial Cell Culture

*E. coli* BL21 and DH10β were transformed with plasmid pDR122 (GFP‐MinD, MinE)^[^ ^11^ ^]^ (kindly provided by Piet de Boer, Case Western University) *E. coli* cells were grown aerobically in Luria–Bertani (LB, Fisher Chemical) medium supplemented with 100 μg mL^−1^ Ampicillin (Fisher Chemical) at 37 °C overnight (ON) from one single colony in LB agar. Then, 100 μL from the ON culture was diluted into 10 mL of fresh medium, BL21 strain was grown for 1.5 and 2.5 h for DH10β at 37 °C and the expression of the recombinant proteins was induced by 25 μm Isopropyl β‐D‐1‐thiogalactopyranoside (IPTG, Generon) for 2 and 3 h (BL21 and DH10β, respectively) at 30 °C in the dark. Bacterial cells were harvested by centrifugation (5600 rpm, 3 min), washed three times with phosphate‐buffered saline, pH 7.4, 0.01 m (PBS, Sigma‐Aldrich), and further diluted in PBS supplemented with glucose 0.1% (VWR Chemicals BDH).

##### Nanofabrication of Nanotopographies

The topography was fabricated on UV‐curable resin OrmoComp. Initially, OrmoComp thin films were produced on glass‐bottom Petri dishes (Ibidi). Prior to that, the Petri dishes were activated with oxygen plasma (Tepla 600) at 50 W for 2 min, to improve the adhesion between polymer and glass. Afterward, a solution of OrmoComp on toluene (150 mg mL^−1^) was spin coated at 3000 rpm for 1 min. The nanocone structures were replicated from PDMS molds, fabricated as previously described^[^ ^14^ ^]^ using a UV lamp (UVASPOT 400/t from Honle) providing 80 mW cm^−2^ for resin curing. The topography of the substrates was imaged by scanning electron microscopy (SEM) using an Auriga FIB‐SEM system (Zeiss).

##### Preparation of SWCNT Dispersion

SWCNTs solid samples (6,5 chirality, ≥95% as carbon nanotubes, 0.78 nm average diameter, Sigma‐Aldrich) were dispersed in Tween 20 saline solution (1% Tween 20 in PBS) (Sigma‐Aldrich) and the suspension was sonicated for 1 h at RT in a bath sonicator (FB15053, Fisherbrand). The dispersed SWCNTs were obtained by several centrifugations at 15 000 rpm for 15 min until no deposit of SWCNT aggregates was detected and good dispersion in the solvent was observed (Figure S4, Supporting Information). The dispersion was further diluted in PBS with glucose 0.1% to a final concentration of 4 μg mL^−1^ SWCNTs and 0.3% Tween 20 and placed in a 20 mL syringe on a one‐channel microfluidic programmable syringe pump (NE‐1002X, New Era Pump Systems Inc.) connected by silicone tube to the microfluidic channel slide (0.8 mm inner diameter, Ibidi). For microfluidic experiments, we first measured the real delivered volume over 5 min to calculate the real flow rate of the microfluidic pump, from which we obtained the shear stress value considering the nominal dimensions of the microfluidic flow chamber. A nominal flow of 5 mL min^−1^ corresponded to a real flow of 3.7 mL min^−1^, and in turn to a shear stress of approximately 12 dyn cm^−2^. We note that a concentration of 4 μg mL^−1^ SWCNTs was low enough to not significantly affect shear stress compared to the control.

A Cary 5000 UV/Vis/NIR Spectrophotometer and a NanoLog 4 HORIBA Spectrofluorometer were used to confirm individual dispersion of SWCNTs (Figure S4, Supporting Information).

##### Microscopy Sample Preparation

For all experiments, either flat and nanostructured OrmoComp surfaces or microfluidic channel slides (50 × 5 mm # 1.5, Ibidi) were coated with 100 μL 0.01% w/v PLL (Sigma Aldrich, P9820, 0.1% w/v) for 10 min, rinsed in MilliQ water and dried with N_2_. We have previously shown that these immobilization conditions do not affect Min oscillations.^[^ ^13a^ ^]^ For flow experiments, the bacterial suspension was deposited inside the channel for 20 min and nonattached cells were removed by rinsing the device three times in PBS with glucose 0.1%.

##### Fluorescence Microscopy

Images were collected through an inverted optical microscope Nikon Eclipse Ti with a total internal reflection objective (60×, 1.49 NA oil immersion Nikon) and an Electron Multiplying CCD camera (EMCCD) (iXon Ultra897, Andor Technology) after passing through the suitable dichroic mirrors (z488rdc or ZT532rdc, Chroma Technology) and additional spectral filters (HQ500 LP and HQ525/50, Chroma Technology, or 641/75 BrightLine, Semrock, respectively). The use of additional lenses resulted in a final magnification of 148×, equivalent to a pixel size of about 108 nm. Samples were excited by laser irradiation (Omicron Laserage Luxx 488 nm at 20 W cm^−2^ for GFP, and Cobolt Samba 532 nm at 7.6 W cm^−2^ for PI). Imaging frames were typically collected at 3 s intervals and 200 ms integration time. To reduce photobleaching, a shutter (SHB05T‐Ø1/2″, Thorlabs) connected to a wave generator (TTi TG330) and synchronized with the EMCCD camera was used to restrict the irradiation that reached the sample only during acquisition. For PI staining experiments, PI was added directly to the sample to a final concentration of 2.0 μm.

##### Data Processing

All data were acquired and processed with Andor Solis software. MinD oscillations along each pole were analyzed by measuring the average fluorescence intensity from region of interest of 4 × 4 pixels at one pole of the bacterium. Data obtained were fitted with a damped sine wave Equation (1) using GraphPad Prism 9.0.

(1) $$Y=A\cdot e^{-K\cdot X}\cdot \text{sin}\frac{2\pi \cdot X}{W}+P$$

where *A* is the maximum amplitude of the wave, *K* is the decay constant, *W* is the wavelength as time taken for a complete cycle, and *P* is the phase shift at the earliest time when *Y* = 0.

Statistical analysis of differences between groups was performed using a two‐tail *t*‐test and considered statistically significant when *p* < 0.05 (*). Data were analyzed using GraphPad Prism 9.0.

## Conflict of Interest

The authors declare no conflict of interest.

## Supporting information

Supplementary Material

Supplementary Material

Supplementary Material

Supplementary Material

## Data Availability

The data that support the findings of this study are available from the corresponding author upon reasonable request.
