# Supplementary material for: Real‐Time Imaging of the Mechanobactericidal Action of Colloidal Nanomaterials and Nanostructured Topographies
Source: Small Sci. 2023 Apr 5;3(5):2300002. doi: 10.1002/smsc.202300002 (PMC11935832; doi:10.1002/smsc.202300002)
Supplement: Supplementary file 1 — Supplementary Material [file SMSC-3-2300002-s001.pdf]

Real-Time Imaging of the Mechanobactericidal Action of Colloidal Nanomaterials and Nanostructured Topographies

Felipe Viela,[1‡] Ingrid V. Ortega,[1 ‡] Jaime J. Hernández,[1] Isabel Rodríguez,[1] Sara Moreno-Da Silva,[1] Alejandro López-Moreno,[1] Emilio M. Pérez,[1] Cristina Flors[1,2]*

[1]Madrid Institute for Advanced Studies in Nanoscience (IMDEA Nanociencia), C/ Faraday 9, Madrid 28049, Spain

[2]Nanobiotechnology Associated Unit CNB-CSIC-IMDEA, C/ Faraday 9, Madrid 28049, Spain

[‡]Equal contribution

[*]To whom correspondence should be addressed at cristina.flors@imdea.org

Supporting Information

FIGURE S1

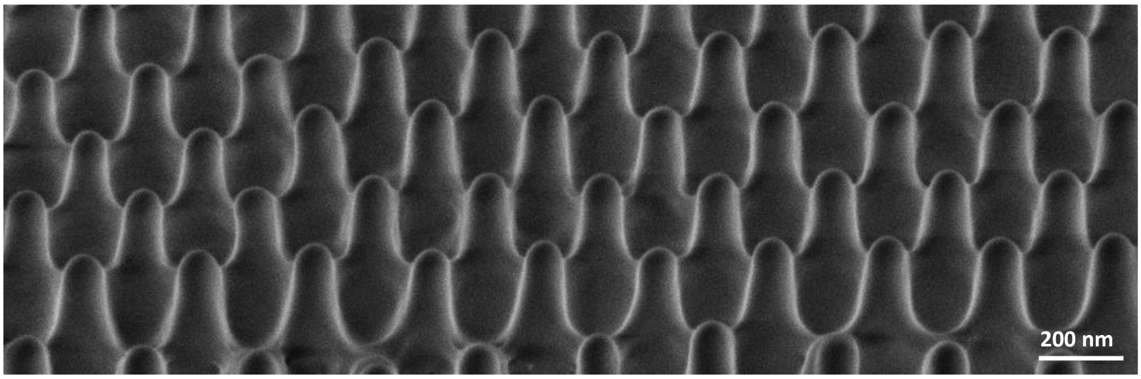

Figure S1. Scanning electron microscopy image of OrmoComp nanostructured topography.

FIGURE S2

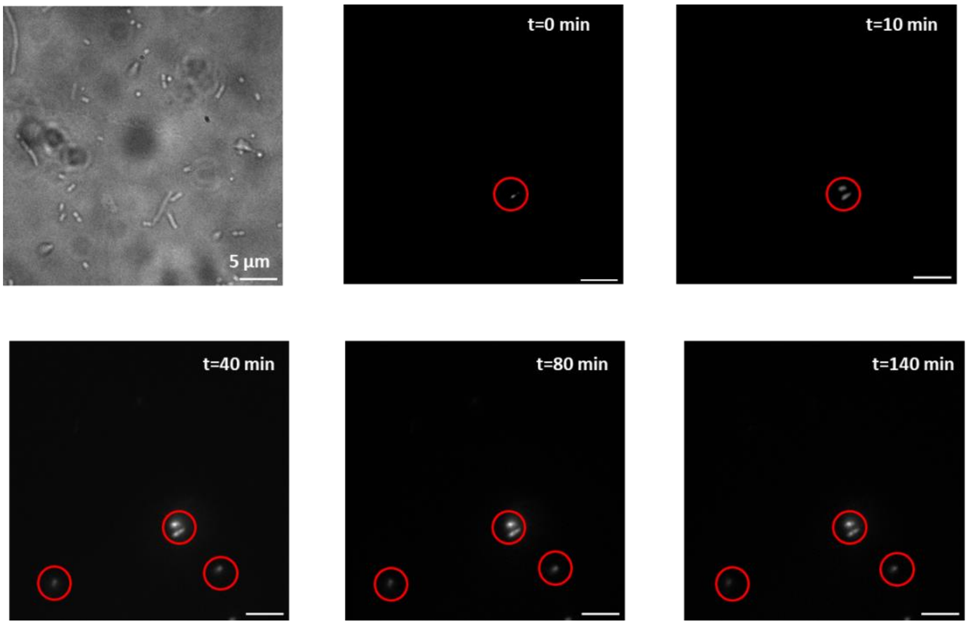

Figure S2. Time-lapse imaging of bacterial death (propidium iodide staining) induced by OrmoComp nanotopographies.

FIGURE S3

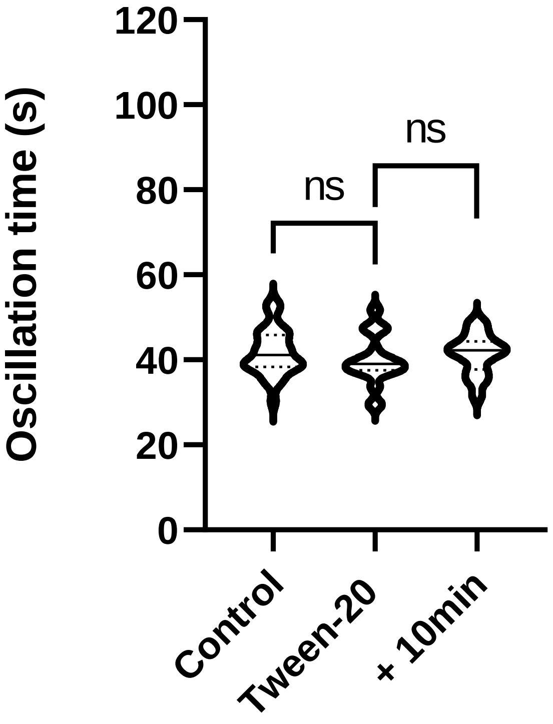

Figure S3. Shear stress of 12 dyn/cm^2 of PBS with 0.3% Tween-20 does not affect Min oscillations significantly.

FIGURE S4

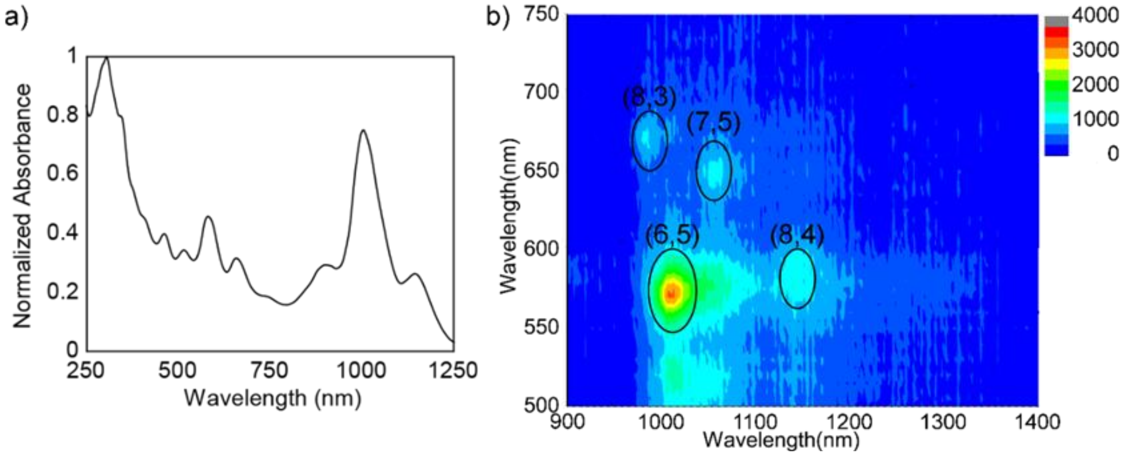

Figure S4. a) Normalized UV-vis-NIR spectrum of (6,5)-SWCNTs (4 µg/ml, PBS, 0.3% Tween-20, 298K). The spectrum is dominated by narrow absorbance bands with a relatively low contribution of scattering, confirming that SWCNTs are individually dispersed; b) PLE intensity map of (6,5)-SWCNTs (4 µg/ml , PBS, 0.3% Tween 20, 298K). Strong PL confirms individualization of (6,5)-SWCNTs. Other SWCNT chiralities are also observed.

FIGURE S5

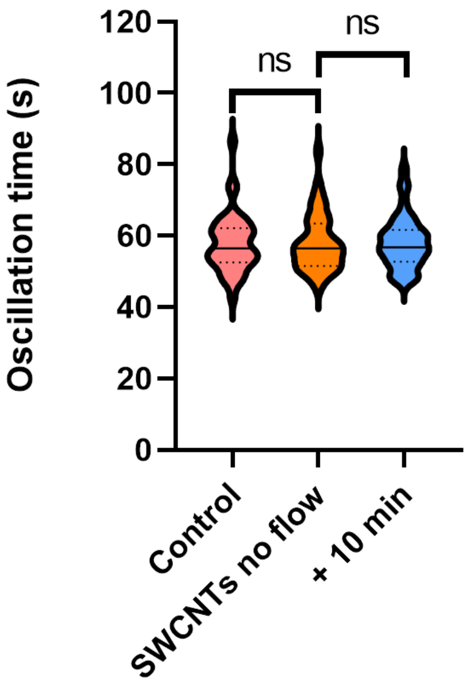

Figure S5. Static incubation with 4µg/ml SWCNTs does not affect Min oscillations significantly.
