# Supplementary figures and images for: Real‐Time Imaging of the Mechanobactericidal Action of Colloidal Nanomaterials and Nanostructured Topographies
Source: Small Sci. 2023 Apr 5;3(5):2300002. doi: 10.1002/smsc.202300002 (PMC11935832; doi:10.1002/smsc.202300002)

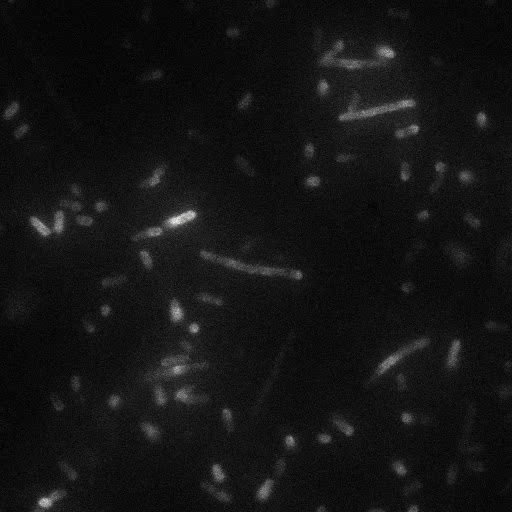

Supplement: Supplementary file 2 — Supplementary Material [file SMSC-3-2300002-s004.gif]

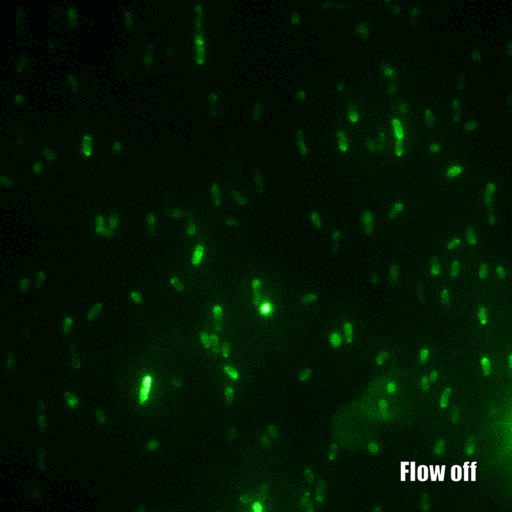

Supplement: Supplementary file 3 — Supplementary Material [file SMSC-3-2300002-s002.gif]

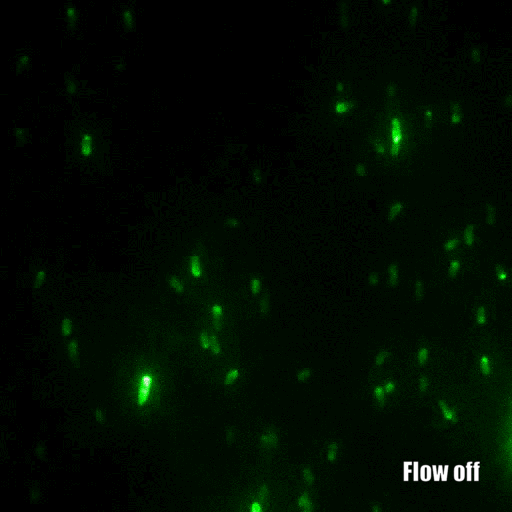

Supplement: Supplementary file 4 — Supplementary Material [file SMSC-3-2300002-s003.gif]
